# A Bayesian Interrupted Time Series framework for evaluating policy change on mental well-being: An application to England’s welfare reform

**DOI:** 10.1016/j.sste.2024.100662

**Published:** 2024-06-11

**Authors:** Connor Gascoigne, Annie Jeffery, Zejing Shao, Sara Geneletti, James B. Kirkbride, Gianluca Baio, Marta Blangiardo

**Affiliations:** a MRC Centre for Environment and Health, Department of Epidemiology and Biostatistics, School of Medicine, Imperial College London, London, UK; b Division of Psychiatry, University College London, Psylife Group, London, UK; c Department of Statistical Science, University College London, London, UK; d Department of Statistics, London School of Economics and Political Science, London, UK

**Keywords:** Bayesian hierarchical model, Interrupted time series, Mental well-being, Policy evaluation, Spatial random effect

## Abstract

Factors contributing to social inequalities are associated with negative mental health outcomes and disparities in mental well-being. We propose a Bayesian hierarchical controlled interrupted time series to evaluate the impact of policies on population well-being whilst accounting for spatial and temporal patterns. Using data from the UKs Household Longitudinal Study, we apply this framework to evaluate the impact of the UKs welfare reform implemented in the 2010s on the mental health of the participants, measured using the GHQ-12 index. Our findings indicate that the reform led to a 2.36% (95% CrI: 0.57%–4.37%) increase in the national GHQ-12 index in the exposed group, after adjustment for the control group. Moreover, the geographical areas that experienced the largest increase in the GHQ-12 index are from more disadvantage backgrounds than affluent backgrounds.

## 1 Introduction

In recent years, the importance of mental health and well-being has become more apparent, with the World Health Organisation directly promoting it in target 3.4 of its Sustainable Development Goals (UN, 2022). Social inequalities can be both a basis for, and result of, poor mental health (FPH, 2016). For instance, people suffering from mental ill health are more likely to have insecure employment and housing (Butterworth et al., 2012; Milner et al., 2014); at the same time individuals experiencing social and financial difficulties are at a high risk of mental ill health (Yu, 2018; Ribeiro et al., 2017). In this context, changes in policy that directly target those who are considered more socially disadvantaged can have a profound effect on their mental well-being. It is crucial to ensure such changes do not worsen their situation, but rather aim to mitigate any existing disparities.

In the context of public health and policy evaluation settings, we often need to rely on observational data. However, a key limitation for observational studies is fully and properly accounting for confounding. One method of capturing any unmeasured confounding is to include temporal and spatial trends as they can be seen as a proxy for social, health and environmental variables that cannot be explicitly included in the model. In addition, capturing spatial variations is vital as there are differences in the quality of health services (Corris et al., 2020; Ellis and Fry, 2010), and wealth and economy (Forth, 2021; Marmot, 2020; IFS, 2021) which can obscure the true effect of policies. In this paper, we propose an interrupted time series (ITS) model within a Bayesian hierarchical framework to examine the effects of policy change on mental health outcomes. We account for spatial and (non-linear) temporal trends, as well as evaluate inequalities indexed by deprivation and ethnicity. As an illustrative example we evaluate the effect of the United Kingdom (UKs) welfare policy reform known as Universal Credit (UC) on self-reported mental well-being.

UC was introduced in the early 2010s as part of a process of welfare reforms initiated by the coalition government led by then Prime Minister David Cameron of the Conservative Party, to replace six separate welfare benefits (i.e., Child Tax Credit, Housing Benefit, Income Support, income-based Jobseeker’s Allowance, income-related Employment and Support Allowance, and Working Tax Credit) by a single unified welfare benefit (Parliament, 2020). The process was intended to be simpler and facilitate access and receipt of welfare according to need. However, the implementation of UC was marred by controversies, including a lengthy delay in payment and increased sanctions, meaning that individuals received reduced amounts or no amount of welfare support at all, sometimes for prolonged periods (Craig and Katikireddi, 2020; Cheetham et al., 2022; Mahase, 2015). To the best of our knowledge, there has only been one longitudinal study that evaluated the early relationship between UC and mental well-being which considered the effect of when UC was ‘introduced’ to a local authority (i.e., at least one person started receiving UC) and showed how those who were unemployed suffered a disproportionate decline in self-reported mental health (Wickham et al., 2020). Whilst this work provided important insights into the difference between employed and unemployed participants accounting for several influential demographic confounders, it did not consider any differences due to geographical (spatial) location, which, in the context of mental health outcomes, are well established (Faris and Dunham, 1939).

The remainder of the paper is organised as follows: in Section 2, we outline the selected participants, formally define how we measure the policy, the outcome, the exposed and control population, and any confounding variables; we then present the statistical model and describe the implementation strategy. In Section 3, we include the results of the proposed model broken down into the temporal and spatial trends and the confounders. Moreover, we include the results for an index use to quantify the overall change in the exposure group (adjusted for the control group) and a sensitivity analysis. Finally, Section 4 concludes the paper with a discussion.

## 2 Methods

### 2.1 Study population

We consider individual-level mental health outcomes as yearly self-reported responses about psychological distress from Understanding Society (UKHLS, 2022). The UKHLS dataset is a representative longitudinal panel survey based on a stratified two-stage cluster sampling design. The sample consists of approximately 40,000 households that were first interviewed in 2009 and have been followed since in waves that span roughly three years. Specifically, we have data on years 2009–2021. Interviews are either conducted face-to-face by trained individuals, via telephone, or can be completed online. The purpose of the UKHLS is to provide insight into a range of different topics that include work, education, income, family, social life, and health (including mental well-being).

For full details of the sampling design, see UKHLS (2012). Briefly, the stratification was based on variables relating to the UKs regions (12 in total; 10 in England with London additionally split into inner and outer, Scotland and Wales), social class (3 based on non-manual occupations), population density (3 approximately equal), and ethnic minority (ordering based on proportion on non-white population). In total there are 108 stratums based on the 2001 census. With the stratum defined, the first stage of sampling is to select the primary sampling units (PSUs) which we refer to as clusters from hereon. The clusters are defined by postcode sectors (or groups of postcode sectors if fewer than 500 addresses are in the section) from the small user postcode address file. In the second stage, a fixed number (approximately 18) of addresses are selected by equal probability sampling from a list of all addresses in each cluster.

### 2.2 Selected participants

We included individuals who were of a working age (16–64 years old DWP (2022a)) and had information on (i) employment status, (ii) Lower Layer Super Output Area (LSOA) of residence (a total of 32,844 administrative geographies comprising of 400–1200 individuals in order to capture the potential role of neighbourhood characteristics in England defined by the Office for National Statistics (ONS, 2011a)), (iii) the outcome (psychological distress) and (iv) confounding variables (introduced below). We excluded all individuals who did not reside within England (i.e., Scotland, Wales, and Northern Ireland) to combine the UKHLS data with the Index of Multiple Deprivation (IMD; 2007 IMD, 2010 IMD, 2015 IMD, 2019 IMD), an established measure of relative social deprivation for small areas. We excluded people who reported life-time sickness or a disability as they would not qualify for UC, while at the same time would be more likely to experience mental ill-health (Wickham et al., 2020).

### 2.3 Measured outcomes

We measured mental ill health using the 12-item General Health Questionnaire (GHQ-12), a series of non-leading questions that gives an insight into an individual’s general psychological distress over the previous few weeks (Gnambs and Staufenbiel, 2018), and is used to screen for general (non-psychotic) mental health problems. The GHQ-12 is widely used and has been validated in a large survey by the World Health Organisation (WHO; Goldberg et al., 1997). The respondents answer 12 questions, each on a scale of 0–3 mapping onto “non-distress” to “distress”. We sum over these responses to give the GHQ-12 score which can range from 0 (least psychological distress) to 36 (most psychological distress).

### 2.4 Exposure

It is not possible to obtain individual information on the recipient of UC which could be linked to the UKHLS. As UC recipients are typically unemployed, and similarly to Wickham et al. (2020) we defined the exposure based on each individual’s yearly self-reported answer to the employment status question in the UKHLS survey. The exposed population is formed by those who responded as ‘Unemployed’, while anything else, except for “life-time sick or disabled”, was considered in the control population.

### 2.5 Intervention — contextual awareness to universal credit

The policy was rolled out at different times in different Lower Tier Local Authorities (LTLAs; a total of 309 administrative geographies in England defined by the Office for National Statistics (ONS, 2021)), and the transition of individuals from the previous welfare system to UC was performed at different rates. This poses an additional challenge, as there is no way of knowing for certain when an individual started receiving UC. Hence, we defined the intervention in terms of “contextual awareness”. More specifically, let us consider an individual receiving the legacy welfare; we assume that they become aware of others within their LTLA of residency who have already transitioned onto UC. Whilst they might not be on UC themselves, the anticipation of their impending transition has the potential to cause them psychological distress as they become aware of the issues surrounding UC (e.g. well documented lengthy delays and increased sanctions as reported by numerous media outlets). Whilst we cannot be sure of an individual’s awareness to UC, we stipulate that if enough people within the LTLA are on UC, then the LTLA is defined to have become “contextually aware” of UC and the intervention has begun.

To obtain a measure of contextual awareness of UC we used monthly statistics from the Department of Work and Pensions (DWP; DWP, 2022b) based on monthly totals of the number of people in each LTLA registered to receive UC. Once an LTLA reaches the 25% threshold, we assume that the intervention has started. We acknowledge that the choice of threshold percentage is arbitrary, and the definition of the start of the intervention is dependent upon this. Consequently, we performed a sensitivity analysis to see how robust the results are to difference in changes to the definition of intervention and compared the results using the same definition as in Wickham et al. (2020) (an LTLA is introduced to UC when at least one person is receiving UC).

### 2.6 Confounders

As individual-level confounders, we considered each participant’s age, education level, ethnicity, relationship status, and sex. These are common choices to account for the demographic and socioeconomic dimensions (Barnes and Bates, 2017; Zuccotti and O’Reilly, 2019; Grund-ström et al., 2021; McCloud et al., 2023), and are readily available responses in the UKHLS. We also included two area-level confounders at the LSOA level: (i) social deprivation, measured by the 2007, 2010, 2015 and 2019 IMDs (2007 IMD, 2010 IMD, 2015 IMD, 2019 IMD), and (ii) diversity defined as the non-white proportion of the population (including Gypsy and Roma) as reported in the 2001, 2011 and 2021 census (2001 Ethnic proportions, 2011 Ethnic proportions, 2021 Ethnic proportions).

For deprivation, we ranked each LSOA based on their IMD score and grouped them into deciles (1 = most deprived, 10 = least deprived). For diversity, we grouped the LSOAs into four categories, 1 (least diverse) to 4 (most diverse), with cut points at half the national average of non-white population, the national average, and two-fold the national average. As the study period spanned three censuses, we had three national averages 12%, 18%, and 24%, that we considered for the 2009–2010, 2011–2020 and 2021, respectively.

### 2.7 Statistical model

To evaluate the impact of universal credit on mental health, we propose a controlled Interrupted Time Series (ITS) within a Bayesian hierarchical framework. A typical ITS design is used to measure an outcome over an interruption (the intervention or treatment) and compare differences in the level and the temporal trend from before-to-after (Wagner et al., 2002; Penfold and Zhang, 2013). ITS designs have been used to evaluate policies on alcohol (Humphreys et al., 2013) and smoking (Gasparrini et al., 2009) as well as changes in gun violence after police killings (Larson et al., 2023) and hospitalisations after airport closures (Pearson et al., 2016).

For each individual, *i*, in month, *t*, the continuous score for psychological distress is modelled as

$$y_{it}\sim \text{Normal}\left(\mu_{it},\sigma^{2}\right)$$

where *μ*_*it*_ is the underlying mean function. The ITS model with controls can be written as

(1) $$\begin{array}{l} \mu_{it}=\beta_{0}+\;{\text{month}}_{s(i)}\beta_{1}+\;{\text{intervention}}_{ts(i)}\beta_{2}+\;{\text{month}}_{s(i)}^{+}\beta_{3}+\\ \;{\text{exposed}}_{it}\beta_{4}+\left({\text{month}}_{s(i)}\times {\text{exposed}}_{it}\right)\beta_{5}\\ \;+\left(\;{\text{intervention}}_{ts(i)}\times \;{\text{exposed}}_{it}\right)\beta_{6}+\left(\;{\text{month}}_{s(i)}^{+}\times \;{\text{exposed}}_{it}\right)\beta_{7}+\\ \;{\text{age}}_{it}\beta_{8}+\;{\text{education}}_{it}\beta_{9}+\;{\text{ethnicity}}_{i}\beta_{10}+\;{\text{relationship}}_{it}\beta_{11}\\ \;+{\text{sex}}_{i}\beta_{12}+\\ \;{\text{deprivation}}_{ts(i)}\beta_{13}+\;{\text{diversity}}_{ts(i)}\beta_{14}+\\ \;\gamma_{t}+\delta_{s(i)}+\psi_{p(i)}+\kappa_{k(i)}+\epsilon_{i}, \end{array}$$

where the notation *s*(*i*), *p*(*i*), and *k*(*i*) are read as “the LSOA within which individual *i* resides”, “the stratum within individual *i* is classified”, and “the cluster within which individual *i* is sampled from”, respectively.

A typical ITS model includes a baseline (or intercept, i.e., *β*_0_), an overall linear time trend (i.e., month_*s*(*i*)_*β*_1_), the immediate effect of the intervention represented as a dichotomous variable (i.e., intervention_*ts*(*i*)_*β*_2_), and an additional linear term to estimate any change in the temporal trend after the intervention occurs (i.e., ${\text{month}}_{s(i)}^{+}\beta_{3}$) (Bernal et al., 2017). Additionally in our model we include an exp^*s*(^o^*i*)^sure variable, which distinguishes exposed individuals from the control ones, as well as allowing for interactions between the ITS terms and the exposure group. In Eq. (1), {*β*_4_, *β*_5_, *β*_6_, *β*_7_} estimate the difference between the control and exposed group for the baseline, overall linear trend, immediate effect, and sustained effect of the intervention, respectively. The effects of individual and area level confounders are estimated through {*β*_8_, …, *β*_14_}. The terms, *γ*_*t*_ and *δ*_*s*(*i*)_ are random effects used to account for any residual variation in time and space, respectively, not captured by the fixed effects. The temporal random effect is over months and the spatial random effect is over the LSOAs. The UKHLS is based on a stratified two-staged cluster sampling design. To account for the complex survey design, we follow the recommendations in Chapter 11 of Lohr (2010) who say the design can be considered in the modelling framework with the inclusion of parameters for the stratification and clustering. In our context, these are the random effects *ψ*_*p*(*i*)_ and *κ*_*k*(*i*)_ to account for the stratification and clustering, respectively. Finally, as we have repeated measurements for each individual, we include an individual-specific random effect, *ϵ*_*i*_, which captures the response behaviours, i.e., method (in-person, online, or telephone) and punctuality (earlier or later in the wave).

### 2.8 Prior specification

As we are framed in a Bayesian approach, we need to specify prior distributions on all the parameters, as described below.

#### 2.8.1 Fixed effects

On the intercept, we specified a weakly informative normal prior *β*_0_ ~ Normal (0, +∞). On the regression parameters we specified weakly-informative, normal prior as *β*_1_, …, *β*_14_ ~ Normal (0, *σ*2 = 1000).

#### 2.8.2 Random effect

For the stratification, cluster and individual random effects, we use the following zero mean Gaussian distributions for their priors,

$$\psi_{p(i)}\sim \text{Normal}\left(0,\sigma_{\psi }^{2}\right),\;\kappa_{k(i)}\sim \text{Normal}\left(0,\sigma_{\kappa }^{2}\right),\;\epsilon_{i}\sim \text{Normal}\left(0,\sigma_{\epsilon }^{2}\right).$$

The residual temporal variation was captured through a first order random walk (RW1) model,

$$f\left(\gamma_{t}\right)|\sigma_{\gamma }^{2}\propto \sigma_{\gamma }^{-(T-1)}\exp\left(-\frac{\sigma_{\gamma }^{-2}}{2}\sum_{t=1}^{T-1}{\left(\gamma_{t+1}-\gamma_{t}\right)}^{2}\right).$$

The spatial random effect, *δ*_*s*(*i*)_, is a weighted average of a structured and unstructured component, which ensures enough flexibility to provide local and global smoothing in a data driven manner (Riebler et al., 2016),

$$\delta_{s(i)}=\sigma_{\delta }\left(\sqrt{\phi }u_{s(i)}^{*}+\sqrt{1-\phi }v_{s(i)}^{*}\right),$$

where $u_{s(i)}^{*}$ and $v_{s(i)}^{*}$ are standardised versions of the structured and unstructured components, respectively, to have variance equal to one, *ϕ* ∈ [0, 1] is the mixing parameter which attributes how much of the residual spatial variation, $\sigma_{\delta }^{2}$, is due to the structured (*ϕ* = 1) or the unstructured components (*ϕ* ^*δ*^ = 0).

#### 2.8.3 Hyperparameters

We specified penalised complexity (PC) priors (Simpson et al., 2017) on standard deviation and mixing parameter components. A PC prior for a given model component is specified through Pr (model parameters > *U*) = *p* where *U* is an appropriate upper bound for the distribution of the parameter and *p* is the probability of the model parameter being in the upper bound.

All the standard deviations parameters (the stratification, $\sigma_{\psi }^{2}$, the clustering, $\sigma_{K}^{2}$, the individual, $\sigma_{\epsilon }^{2}$, the temporal, $\sigma_{\gamma }^{2}$, and the spatial, $\sigma_{\delta }^{2}$, parameters, respectively) have a PC prior where *U* = 1 and *p* = 0.01. Additionally, the spatial effect mixing parameter, *ϕ*, has a Pr (*ϕ* > 1/2) = 2/3 PC prior. The larger *p* for the mixing parameter reflects the prior belief for more of the spatial variation to be described by the unstructured spatial effect.

We fitted the ITS model within a Bayesian hierarchical frame-work using Integrated Nested Laplace Approximation (INLA), as implemented in the R-INLA package (Rue et al., 2009).

### 2.9 Standardised percentage change

To describe the overall impact of UC on mental well-being in the exposed population compared to the control one, we used a measure of standardised (percentage) change. Generally speaking, this measure estimates the change in the probability of experiencing the outcome in the exposed population before and after the intervention, while adjusting for what happens in the controls during the same period.

In the context of our study, and dropping all the indices for simplicity, let E = Exposed, C = Control, A = all years in the study period after the intervention, and B = all years in the study before the intervention. The average score of psychological distress in the exposed population after experiencing contextual awareness to UC is defined as

$$p^{\text{EA}}=\mu^{\text{EA}},$$

where the superscript EA indicates this is the average of the posterior distribution of the linear predictor for all exposed individuals after the intervention only. The average of the posterior of the linear predictor for the exposed population before the intervention is defined as

$${\tilde{p}}^{\text{EB}}=\mu^{\text{EB}}\frac{\mu^{\text{CA}}}{\mu^{\text{CB}}}.$$

where we include the term $\frac{\mu^{\text{CA}}}{\mu^{\text{CB}}}$ to adjust for anything that might have impacted the outcome at population level in the period after vs before the implementation of the policy. Hence, we used the notation$\tilde{p}$ to indicate that this is a standardised score. If $\frac{\mu^{\text{CA}}}{\mu^{\text{CB}}}$ is above 1, the average score of mental distress before the policy implementation for the exposed groups will be inflated to account for the fact that a change after/before is also seen in the control group, hence the effect of the policy implementation will be smaller than if considering directly the difference in distress before and after the policy implementation in the exposed population. A similar approach was used for evaluating health risks from the opening of municipal waste incinerators (Freni-Sterrantino et al., 2019). Finally, we define

(2) $$\rho =\left(p^{\text{EA}}-{\tilde{p}}^{\text{EB}}\right)/{\tilde{p}}^{\text{EB}}$$

as the standardised change, where *ρ* > 0 indicates an increase in the average score of psychological distress following UC. As we use the full posterior distribution of the linear predictors, we derive a corresponding posterior distribution for *ρ*.

In the following results section, we explore the range of termed profiles and for each, presented the posterior medians and 95% Credible Interval (CrI).

Sections 1 to 4 in the Supplementary material contain additional information on the methods section organised as follows: Section 1 presents a flow diagram and sample characteristic table on the selected participants. Section 2 includes a table of all twelve questions form the GHQ-12 questionnaire. Section 3 provides additional details on the specification of the confounders. Finally, Section 4 presents the results of a pre-analysis on model selection for the specification of the random effects.

## 3 Results

After the selection process, we had 380 378 observations from 47 555 distinct individuals followed from 2009–2021 from the UKHLS survey data. Of those individuals, the average age was 40, there was 53.80% female, 27.40% of the non-white ethnicity, and 15.30% were unemployed at some time during the study period.

### 3.1 ITS parameters

Table 1 presents the posterior mean, 95% credible intervals, and probability of exceeding zero displayed as a percentage for the ITS parameters for the controls and exposed population as well as their difference. The difference in the baseline, *β*_4_, is substantial and provides notable evidence that the exposed population has, on average, a higher GHQ-12 score at the start of the study period. The difference in the immediate effect of the intervention, *β*_6_, is positive with a high exceedance, 99.7%, indicating substantial evidence that the exposed population had a larger increase in the GHQ-12 after a contextual awareness to UC when compared to the control population.

The parameters for the difference in the overall linear temporal trend, *β*_5_, and the lasting impact of the intervention, *β*_7_, are differences-in-differences, i.e., they are rates of change. For these parameters, a positive value is a faster rate of change, and a negative value is a slower rate of change. These parameters do not give an indication of the direction of the change (i.e., increasing, decreasing or constant), only whether one temporal trend in increasing or decreasing faster, slower or at the same rate. Therefore, to fully interpret them, we need to consider the respective parameters for the control and exposed populations alongside the difference parameters.

First, we consider the overall linear temporal trend for the control, *β*_1_, and exposed, *β*_1_ + *β*_5_, populations and their difference, *β*_5_. As the overall linear temporal trend for the control and exposed populations are both positive with high exceedance, all over 80%, the positive value for the difference indicates that the GHQ-12 scores for the exposed population are increasing faster during the study period than the scores of the control population.

Now we consider the change in the linear temporal trend after the policy implementation for the control, *β*_3_, and exposed, *β*_3_ + *β*_7_, population and their difference, *β*_7_. All three parameters are either centred around, or extremely close to, zero and are characterised by high uncertainty. Therefore, this suggests that there is not strong evidence of a sustained effect of the intervention.

**Fig. 1:**
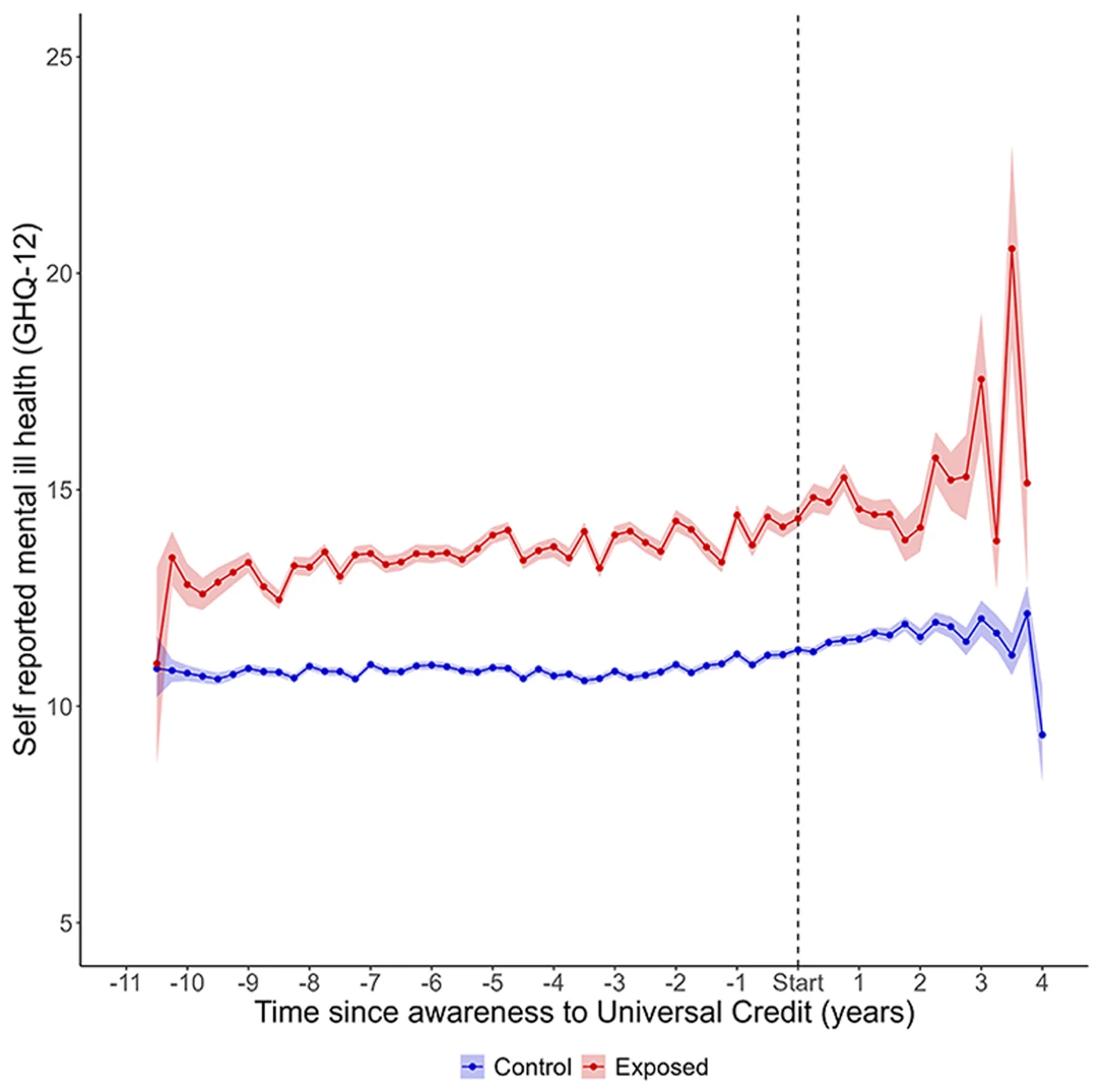
National average GHQ-12 score for exposed (red) and control (blue) populations. The solid line represents the median value of the posterior distribution, and the shaded region is the 95% Credible Interval.

Fig. 1 shows the posterior distribution of the national average GHQ-12 score for the control and exposed population over the entire study period. The results in Table 1 and their interpretations are reflected in Fig. 1. Clearly, there is an immediate difference between the control and exposed populations, and this is captured in the difference between baseline parameter, *β*_4_. Over the entire study period, a steeper positive temporal trend for the exposed population when compared to the control population is seen, and this is reflected by the positive *β*_5_. The gradient of the exposed population at the point before moving closer to the policy implementation (‘Start’ line) is a steeper positive one in comparison to the same part of the control population. This reflects the positive parameter for the difference in immediate effect of the intervention, *β*_6_. Finally, as reflected in *β*_3_ and *β*_7_ there is no visible sustained effect of the intervention, with both the exposed and control populations having similar gradients after the ‘Start’ line.

### 3.2 Spatial variability

**Fig. 2:**
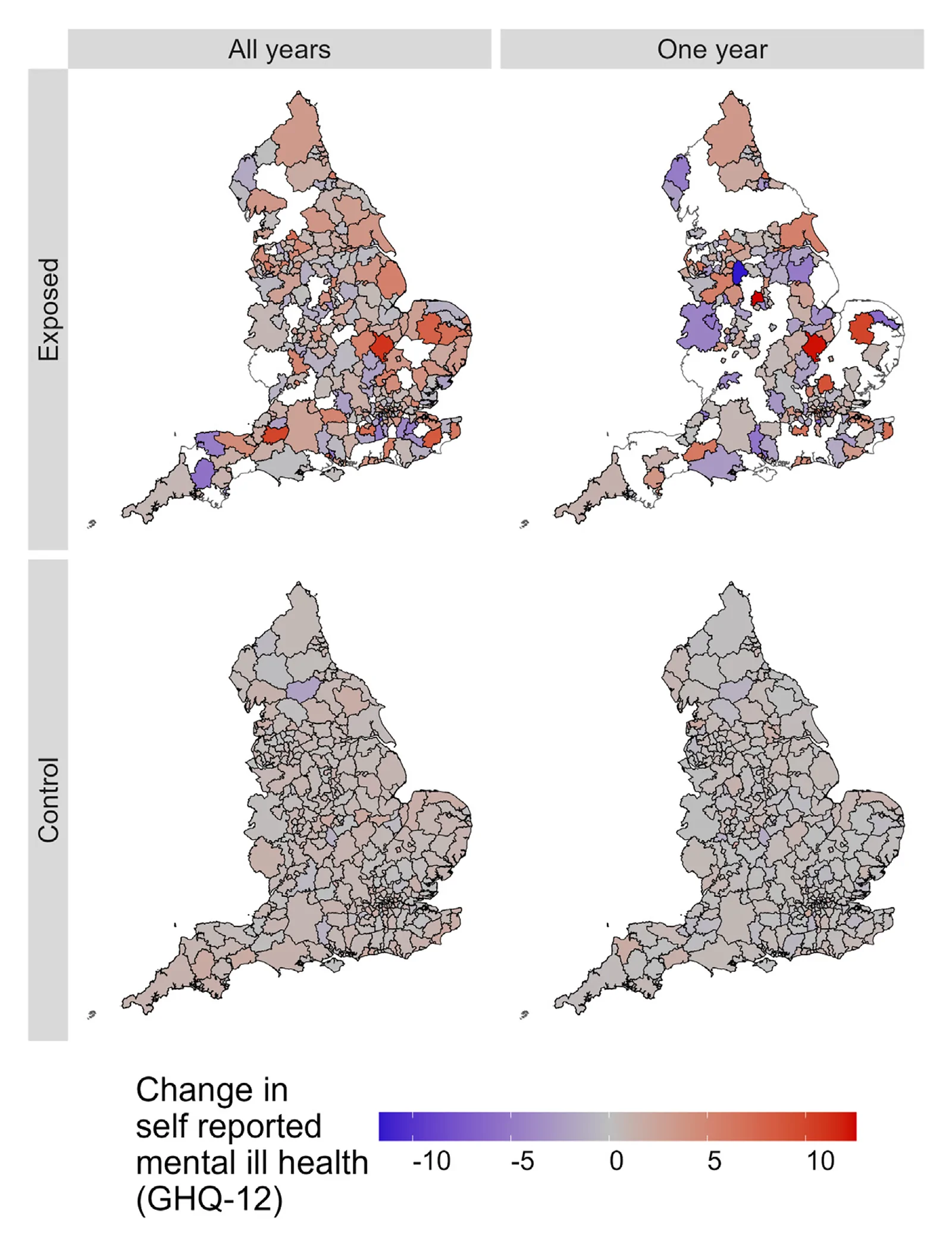
Average GHQ-12 score in the control (bottom row) and exposed (top row) populations before-and-after a contextual awareness to Universal Credit for each Lower Tier Local Authority (LTLA) under two different time frames. The first time frame (left-hand column) shows the average score over all years before-and-after the intervention, while the second time frame (right-hand column) shows the average score over one year before-and-after the intervention. The white colour represents LTLAs where there were no observations either before and/or after the intervention for the specific exposure group/time frame combination.

Fig. 2 presents the difference between the GHQ-12 score before and after the intervention for the control and exposed populations mapped at the LTLA level for two different time frames. The left-hand column shows the difference for all years before and all years after the intervention, and the right-hand column shows the difference restricted to the year before and after the intervention.

For both time frames, the maps of the control population (bottom) are mainly grey indicating, as expected, that the intervention does little to change the average self-reported GHQ-12 scores for those not unemployed. In contrast, the exposed population (top) show greater variability, being dominated by the red colour, indicating higher psychological distress after the introduction of the policy. Additionally, the control population does not show any distinctive change when considering all years in comparison to the year before–after the implementation of the policy. However, the exposed population shows higher increases in average GHQ-12 score when considering all years in comparison to the year before–after the policy implementation.

### 3.3 Confounder results

**Fig. 3:**
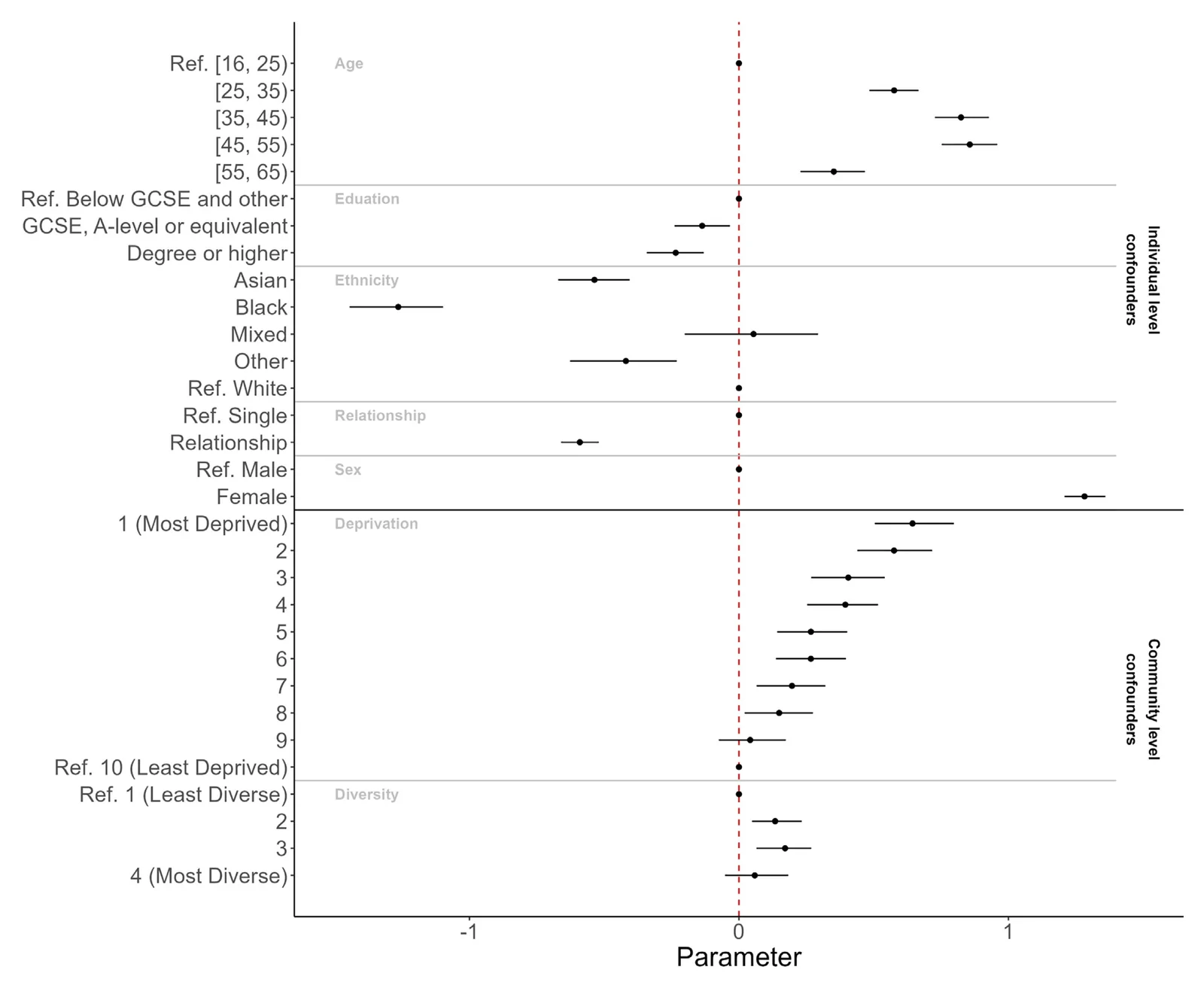
Estimates and 95% credible intervals for the regression parameters related to the individual and community level confounders. A negative value indicates a reduction in the GHQ-12 score with respect to the reference category and a positive value indicates an increase.

Fig. 3 presents the parameters and their associated uncertainty for the individual and community level confounders. The highest GHQ scores are seen for [45, 55) age group, compared to the reference ([16, 25)). There is a clear effect of education, with higher education associated to lower psychological distress. Additionally, being in a relationship is associated to lower GHQ-12 scores compared to being single, while higher levels of the GHQ-12 score are seen for females. Regarding the community characteristics, moving from less deprived to a more deprived area is associated with an increase in GHQ-12 score. There is no clear pattern for diversity, with all the areas characterised by a percentage of non-white population above the national average seeing an increase in psychological distress. For all ethnicities, except for mixed, the GHQ-12 reduces relative to white.

Section 5 of the Supplementary Material presents a table of all the parameters in the final model and plots of the spatial and temporal random effects.

### 3.4 Standardised change

**Fig. 4:**
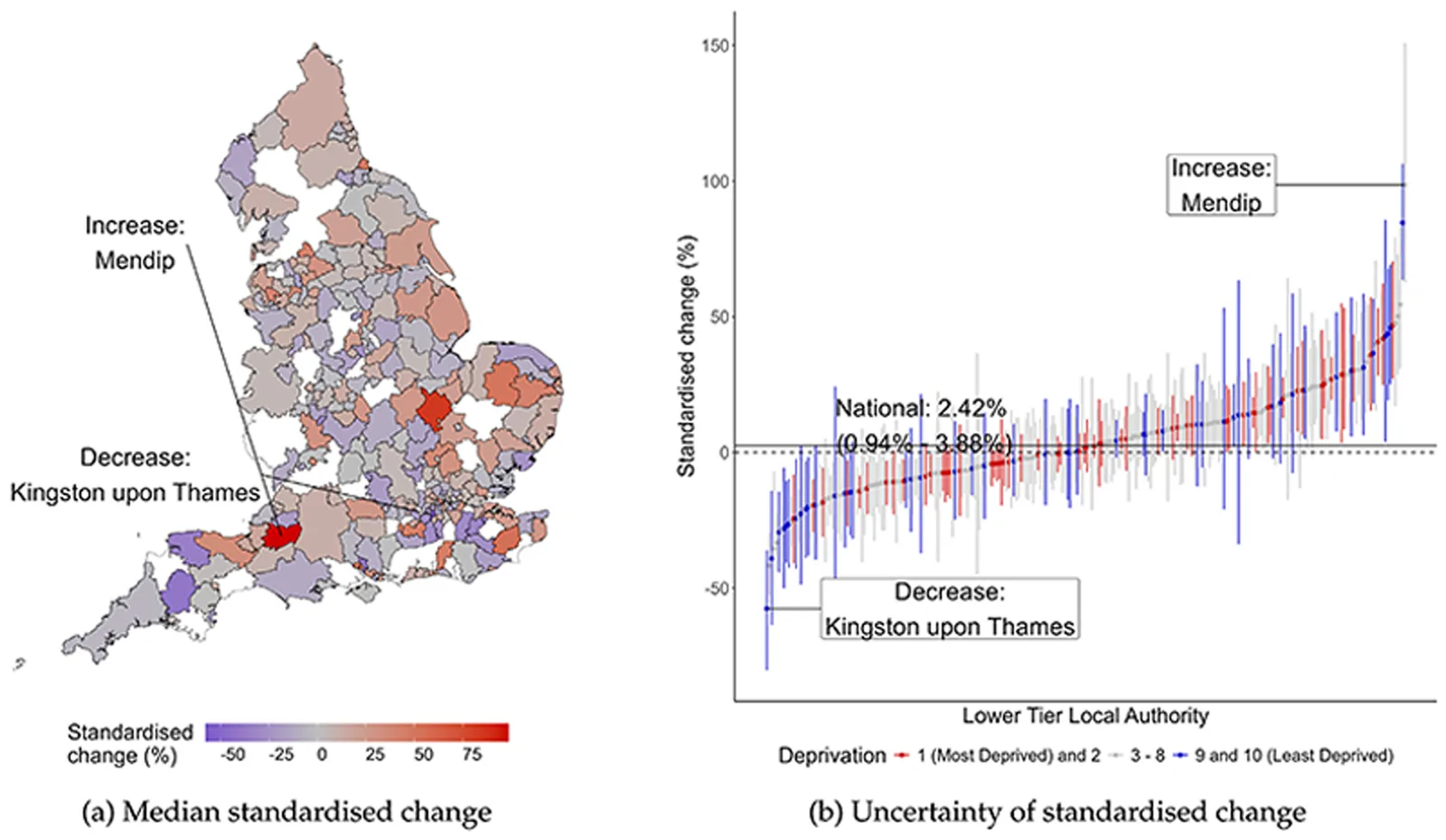
Standardised change due to a contextual awareness of Universal Credit for each Lower Tier Local Authority (LTLA): (a) map showing the distribution by geographical location; (b) 95% Credible Intervals ordered by increases in psychological distress, including the national change. To calculate the standardised change for an LTLA, there needs to be data on GHQ-12 score before and after the policy implementation for both the exposed and control groups in that area. If one of these quantities is missing the standardised change cannot be calculated, consequently appears missing (white in the map).

Fig. 4 shows the standardised change of the GHQ-12 scores due to the intervention (hereafter referred to as standardised change), in the exposed population due to a contextual awareness of UC mapped at the LTLA level. The majority of the LTLAs that see an increase in the standardised change are found along the North-East and North-West coastlines as well as in the midlands. The LTLAs showing no change or decreases in the standardised change are predominantly along the Southern coastline and South-West of London.

Fig. 4(b) presents LTLA-specific standardised change arranged from the largest decrease to the largest increase. The LTLAs are coloured by the deprivation deciles; red indicates the two most deprived deciles, blue the two least deprived deciles and grey those in between. Among the LTLAs in the top 10% decreases in standardised change (left side of the *x*-axis), 42% are from the two least deprived LTLA deciles, whereas 12% were from the two most deprived LTLA deciles. In contrast, among the LTLAs in the top 10% increases in standardised change (right side of the *x*-axis), 25% were from the two least deprived LTLA deciles and 25% were from the two most deprived LTLA deciles. Note the national standardised change reported in the figure, equal to 2.42% (95% CrI: 0.94%–3.88%), providing substantial evidence that a contextual awareness of UC had a negative impact on mental well-being on the unemployed population in England. Overall, 53% of LTLAs saw an increase and 47% saw a decreased in the standardised change.

### 3.5 Sensitivity analysis

**Fig. 5:**
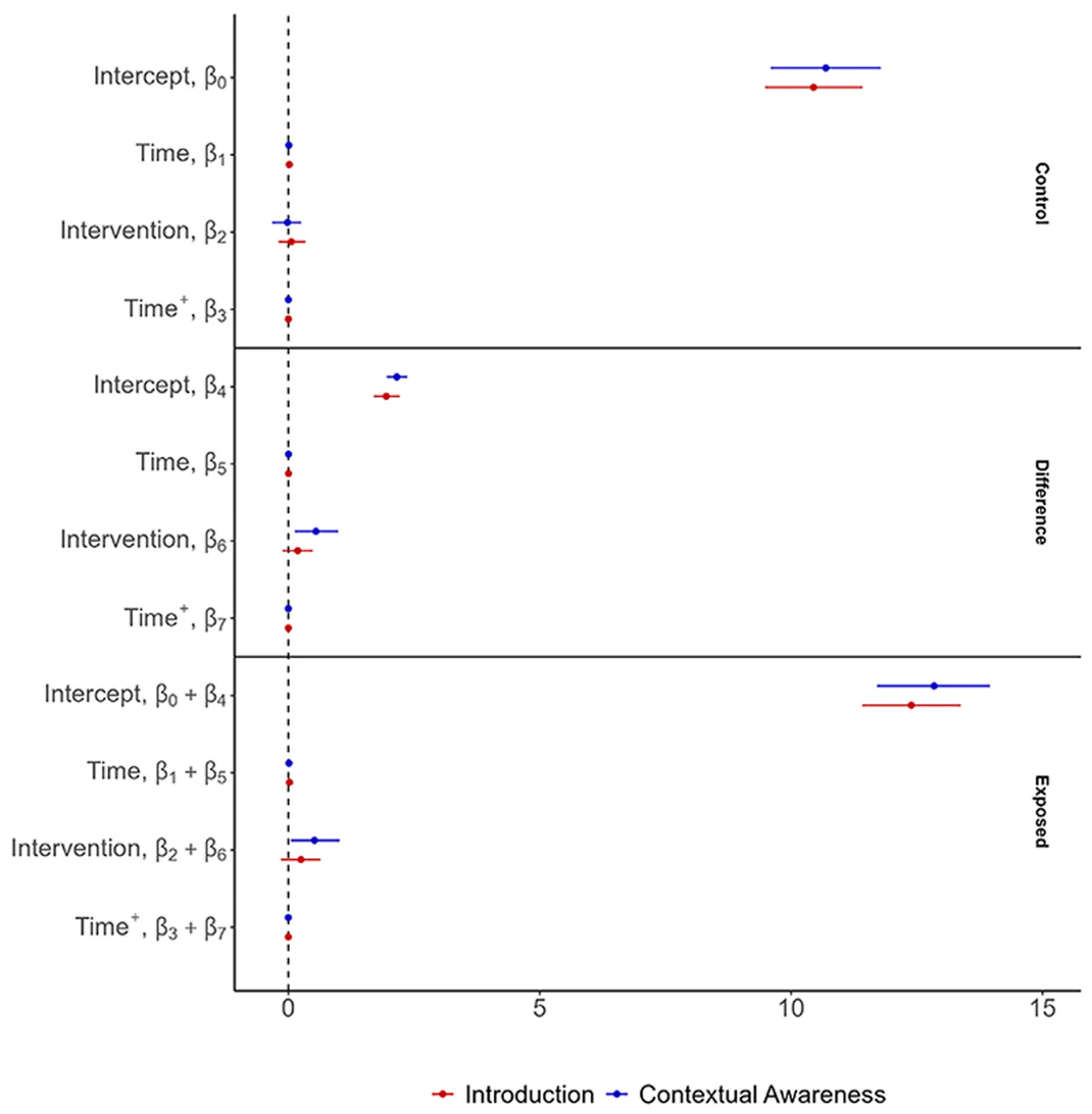
Sensitivity analysis comparing the use of an introduction to Universal Credit and the use of a contextual awareness to Universal Credit as the definition of the intervention starting point.

We performed sensitivity analysis changing the definition of contextual awareness with ‘introduction’, defined as: at least one person in an LTLA is receiving UC. The results are presented in Fig. 5.

Fig. 5 shows the parameters for the baseline (Intercept, *β*_0_, *β*_4_, *β*_0_ + *β*_4_), overall time trend (Time, *β*_1_, *β*_5_, *β*_1_ + *β*_5_), immediate effect of the intervention (Intervention, *β*_2_, *β*_6_, *β*_2_ + *β*_6_) and sustained effect of the intervention (Time^+^, *β*_3_, *β*_7_, *β*_3_ + *β*_7_) under the two definitions for the control population, the difference between the control and exposed population and the exposed population. For each of the parameters the uncertainty intervals overlap highlighting the robustness of the ITS parameters to the definition of when the intervention start being implemented.

Section 6 of the Supplementary Material contains a table of all the parameter estimates from the two model fits in the sensitivity analysis.

## 4 Discussion

In this article, we presented a Bayesian hierarchical interrupted time series with controls, to evaluate policy interventions and demonstrated how this framework can be used to evaluate the impact of welfare reforms on mental ill-health. Our work is subnational in nature, and flexible in accounting for spatial/temporal dependence and individual/community level characteristics; additionally, it naturally allows the construction of additional quantities of interest with their associated uncertainty and provides a deeper insight into the effect of UC on mental well-being than is currently available in the literature. For policy makers, these additional insights are crucial as they provide a wealth of data-driven results on the type of characteristics most negatively affected by the welfare reform. Using this, any similar future policy can be designed and implemented in a manner that ensures those most at risk are protected which is not possible when only considering national level results.

We found that a contextual awareness to UC causes an increase in the GHQ-12 score immediately, which is not sustained in the long term. When considering the spatial relationship between GHQ-12 score and the intervention, we saw that for each LTLA, the exposed population experience larger changes in the average GHQ-12 score before-and-after the intervention in comparison to the control population. Including a random effect at the LSOA level ensures the best adjustment for residual confounding. Due to the survey data being geographically sparse at the LSOA level (53% of LSOAs were seen over the entire study period), we adjusted for the disconnected nature of the non-resolved spatial domain first scaling the precision matrix and then imposing a sum-to-zero constraint on each disconnected components of the spatial domain (Freni-Sterrantino et al., 2018). Consequently, the spatial mixing parameter being close to zero, 0.0449 (95% CrI 0.0377 – 0.0516), reflects the disconnected nature of the spatial domain. Moreover, due to the geographically sparsity at the LSOA level, we presented the spatial results at the LTLA level and even at this level there were some areas which remained missing. In the model we adjusted for a comprehensive set of confounders, both at the individual and area level. The results for ethnicity suggest that all groups, except mixed ethnical background, had a lower GHQ-12 compared to the white population. This may be due to cultural differences in the self-perception, help-seeking, diagnosis, and treatments of mental health (Choudhry et al., 2016), which results in lower self-rating of psychological distress in minoritised ethnic groups. A similar result was presented by Breslau et al. (2017) who saw a reduction in the perceived need for help with mental health disorders in all ethnic minorities compared to white ethnicity. Furthermore, GHQ-12 measures changes in psychological distress in the short term, as the questions compare the last two weeks to how the participant “normally” feels. Therefore, it may not detect high levels of psychological distress in people for whom this is ongoing.

We used the posterior distributions from the model to estimate an index that quantifies the change in the exposed population adjusted for the control for each spatial unit. We saw that nationally the intervention increased the GHQ-12 score for the exposed population, which was mirrored in the majority of LTLAs. Additionally, our results suggest a role of deprivation, with the most deprived communities often seeing the largest increases in GHQ-12 scores. This is in line with the HSE (2017) which reported that in the least deprived areas, 13% of men and 15% of women had a GHQ-12 response that can be classified as ‘poor mental well-being’. When considering the responses from the most deprived areas, this rose to 21% of men and 24% of women.

Our approach works in the same perspective as Wickham et al. (2020) who used a difference-in-difference (DID) design to assess the impact of UC on mental health, adjusting for individual level confounders. A DID only considers one measurement before and after an intervention and does not include a temporal trend. We exploited the natural longitudinal nature of the data specifying an ITS, which we believe is better suited for interventions in time (Wagner et al., 2002; Penfold and Zhang, 2013). In addition to the statistical method and definition of when the intervention begun, we accounted for community level deprivation and diversity as well as residual confounding in time and space. Nevertheless, our results are overall in line with Wickham et al. (2020), which reported a 1.28 (0.61 – 1.95) increase in GHQ-12 score in the exposed due to an introduction to UC. Our equivalent result was the 2.42% (95% CrI: 0.94%–3.88%) standardised change in the exposed GHQ-12 score. Differences can be attributed (i) to methodological aspects (e.g., the type of quasi-experimental design and measured outcome), the covariates included in the model and, (ii) difference in data sets (2009–2018 in Wickham et al. (2020) vs 2009–2021 in our study).

Our method suffers from limitations related to some of the assumptions: (i) the use of unemployment as a proxy for exposure to UC, and (ii) the definition of the start of the intervention. For limitation (i), during 2017–2022, among the people on UC, approximately 40% were in employment (DWP, 2022c). Consequently, while we were forced to use employment as a proxy for exposure to UC as there was insufficient individual level data on UC from the UKHLS, the results for the exposed and control group might be under- and over-estimated, respectively. For limitation (ii), we performed a sensitivity analysis changing the definition from UC awareness to introduction and found the parameters were robust to the definition of the intervention.

To conclude, we believe that the Bayesian hierarchical framework is the natural approach for evaluating the impact of policy interventions at population level, taking advantage of the intrinsic longitudinal nature of the data as well as of spatial and temporal dependencies. The framework we provided is not solely applicable for changes in mental well-being due to UC; for example, the same framework could be used to assess the impact of other policy changes in the UK on mental health outcomes, i.e., the impact of the UK’s 2012 Suicide Prevention Strategy on suicide rates. Alternatively, it can be used more generally on “shocks” in time that affect health outcomes, i.e., the impact of COVID-19. For all scenarios, the framework can be used to provide data-driven recommendations for future policy to ensure there are reduced negative impacts and those most at risk are not disproportionately affected.

## Supplementary Material

Supplementary material related to this article can be found online at https://doi.org/10.1016/j.sste.2024.100662.

Supplementary Material

## Figures and Tables

**Table 1:** Interrupted Time Series parameters for the control, difference and exposed parameters calculated from the posterior distribution of the parameters.

	Parameter (95% Credible interval)	Pr (Parameter 0) x 100
**Control:**		
Intercept, *β*_0_	10.6979 (9.5313, 11.7864)	100.00
Time, *β*_1_	0.0089 (−0.0165, 0.0327)	80.90
Intervention, *β*_2_	−0.0222 (−0.285, 0.2413)	42.50
Time+, *β*_3_	−0.0004 (−0.0019, 0.001)	27.30
**Difference:**		
Intercept, *β*_4_	2.1617 (1.9858, 2.3503)	100.00
Time, *β*_5_	0.0026 (0.0001, 0.0053)	97.80
Intervention, *β*_6_	0.5316 (0.1236, 0.9639)	99.70
Time+, *β*_7_	−0.0011 (−0.002, −0.0003)	0.50
**Exposed:**		
Intercept, *β*_0_ + *β*_4_	12.8423 (11.7108, 13.9713)	100.00
Time, *β*_1_ + *β*5	0.0115 (−0.0142, 0.0355)	86.10
Intervention, *β*_2_ + *β*_6_	0.507 (0.0414, 0.9603)	9 8.20
Time^+^, *β*_3_ + *β*_7_	−0.0015 (−0.0031, 0.0002)	4.30

## Data Availability

From the UKHLS, the main survey is free to download after registration, but for data sets that contain geographical information, special licences are required (UKHLS, 2022). Data on when a LTLA was made contextually aware to UC is free to download from the DWP after registration (DWP, 2022b). Data on the IMD (2007 IMD, 2007; 2010 IMD, 2011b; 2015 IMD, 2015; 2019 IMD, 2019), ethnicity proportions (2001 Ethnic proportions, 2001; 2011 Ethnic proportions, 2022a; 2021 Ethnic proportions, 2022b), and the spatial shapefiles of England (ONS, 2023) are free to access and download from public resources. The full code for implementing the analysis in this paper can be found at https://github.com/connorgascoigne/Bayesian-ITS-for-policy.
