# Supplementary material for: A Bayesian Interrupted Time Series framework for evaluating policy change on mental well-being: An application to England’s welfare reform
Source: Spat Spatiotemporal Epidemiol. Author manuscript; Available in PMC 2026 Oct 24. (PMC7619630; doi:10.1016/j.sste.2024.100662)
Supplement: Supplementary file 1 [file EMS219626-supplement-Supplementary_Material.pdf]

Supplementary Material for: Bayesian Interrupted Time Series for evaluating policy change on mental well-being: an application to England's welfare reform

C. Gascoigne[1*], A. Jeffery[2], Z. Shao[3], S. Geneletti[4], J. B. Kirkbride[2], G. Baio[3], and M. Blangiardo[1]

[1]MRC Centre for Environment and Health, Department of Epidemiology and Biostatistics, School of Medicine, Imperial College London, London, UK
[2]Division of Psychiatry, University College London, Psylife Group, London, UK
[3]Department of Statistical Science, University College London, London, UK

[4]Department of Statistics, London School of Economics and Political Science, London, UK
[*]Corresponding author. Email: c.gascoigne@imperial.ac.uk

1 Selection criteria and sample characteristics

Figure 1 shows a flow chart of the data processing steps including the observations removed from the exclusion criteria and missingness.

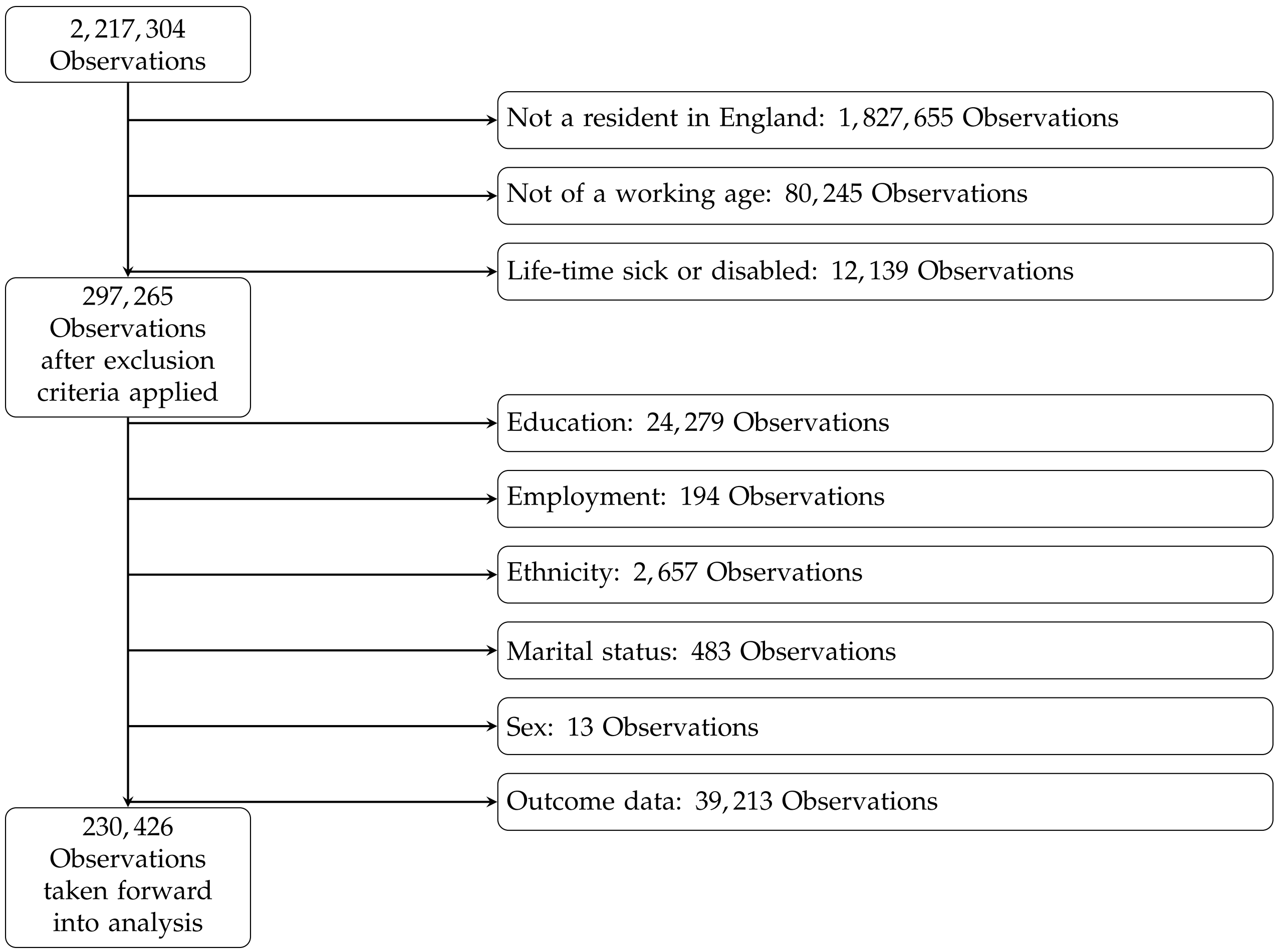

Figure 1: Flowchart of the data processing steps.

Figure 2 shows the difference between the raw prevalence of psychological distress for those who responded to the employment status as lifetime sick or disabled, unemployed (exposed) and all others (control). Not only do those who are lifetime sick or disabled have a separate benefit they receive, they have a vastly different prevalence of psychological distress in comparison to the control and exposed populations. Therefore, we removed these respondents from the data.

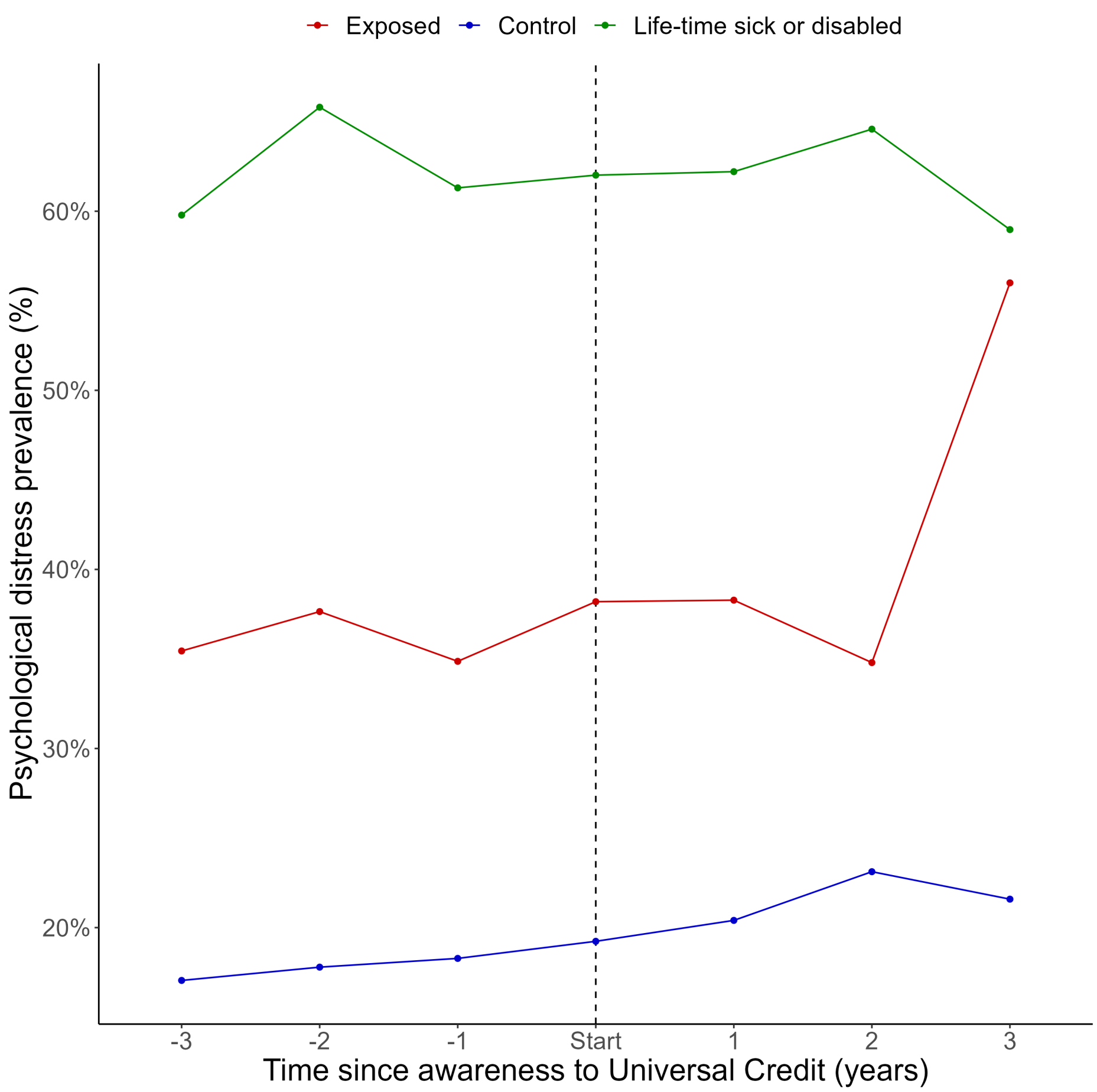

Figure 2: Psychological distress prevalence for the three years before and after a contextual awareness to Universal Credit. The observations are grouped into the exposure grouped as defined by the self-reported response to the employment status question. The red and blue lines are for the exposed and control population, respectively. The green line is for the self-reported 'Life-time sick or disabled' responses to employment status.

After the selection process, there as 230 426 rows of data for 47 555 distinct individuals. Table 1 shows the sample characteristics. The distribution of age, marital status, sex, deprivation, and ethnic mix is (relatively) uniform. The distribution of education and ethnicity is less so. However, the White ethnic group having 72.60% of the population is representative of the UK average in the 2021 census of England and Wales (ONS, 2001b).

Table 1: Sample characteristics after the selection process.

Category	Percentage
**Employment**	
Unemployed	15.31
Not unemployed	84.69
**Age**	
[16, 25)	21.49
[25, 35)	20.38
[35, 45)	21.22
[45, 55)	20.17
[55, 65)	16.74
**Education**	
GCSE, A-level or equivalent	41.26
Degree or higher	36.05
Below GCSE and other	22.69
**Ethnicity**	
Asian	16.44
Black	7.47
Mixed	2.91
Other	4.41
White	68.77
**Relationship**	
Single	43.66
Relationship	56.34
**Sex**	
Male	46.17
Female	53.83
**Deprivation**	
1 (Most Deprived)	10.41
2	11.98
3	10.98
4	10.53
5	10.07
6	10.30
7	9.55
8	9.75
9	8.98
10 (Least Deprived)	7.46
**Diversity**	
1 (Least Diverse)	25.36
2	14.86
3	17.26
4 (Most Diverse)	42.52

2 Outcome

General Health Questionnaire (GHQ) – 12. Scoring rules are as follows: Better than usual = 0; Same as usual = 1; Less than usual = 2; Much less than usual = 3. Sum over all the scores to give a total 0 (no psychological distress) – 36 (maximum psychological distress).

Table 2: The General Health Questionnaire (GHQ) – 12. Respondents using a grade of 0 (Better than usual) – 3 (Much less than usual) in reply where the higher grade, the more distressed.

Question	Response			
	Better than usual	Same as usual	Worse than usual	Much worse than usual
Have you recently been able to concentrate on whatever you're doing?				
Have you recently lost much sleep over worry?				
Have you recently felt that you were playing a useful part in things?				
Have you recently felt capable of making decisions about things?				
Have you recently felt constantly under strain?				
Have you recently felt you couldn't overcome your difficulties?				
Have you recently been able to enjoy your normal day-to-day activities?				
Have you recently been able to face up to problems?				
Have you recently been feeling unhappy or depressed?				
Have you recently been losing confidence in yourself?				
Have you recently been thinking of yourself as a worthless person?				
Have you recently been feeling reasonably happy, all things considered?				

3 Confounders

We used individual (age, education, ethnicity, marital status, and sex) and community level (deprivation and diversity) confounders. The individual level confounders come directly from UKHLS responses, and the community level confounders are derived from the Office for National Statistics (ONS) datasets. A table of the confounders and the breakdown of the categories of each are presented in Table 3

Table 3: Individual and community level confounder categories.

Confounder	Categories	Reference Category
Age	[16, 25), [25, 35), [35, 45), [45, 55), [55, 65)	[16, 25)
Education	Degree or higher, GCSE, A levels or equivalent, Below GCSE or other	Degree or higher
Ethnicity	White, Mixed, Asian, Black, Other	White
Marital status	Single or in a relationship	Single
Sex	Male, Female	Male
Deprivation	1 (Most Deprived), 2, 3, 4, 5, 6, 7, 8, 9, 10 (Least Deprived)	10 (Least Deprived)
Diversity	1 (Least Diverse), 2, 3, 4 (Most Diverse)	4 (Most Diverse)

The community-level confounders were defined at the Lower Layer Super Output Areas (LSOAs), an administration level comprising of 400–1200 individuals with over 33 000 in England, designed to improve the reporting of small area statistics (ONS, 2021). These are updated every ten-years based on the new population estimates from new censuses. The IMD measures relative levels of deprivation across the country at LSOA-level based on 39 separate indicators. It follows an established methodological framework and is widely used in epidemiological research and has releases in 2007, 2010, 2015 and 2019 (2007 IMD, 2010 IMD, 2015 IMD, 2019 IMD) that cover our study period. The diversity community level confounder is derived from LSOA-level ethnic population estimates from the census with the 2001, 2011 and 2021 (2001 Ethnic proportions, 2011 Ethnic proportions, 2021 Ethnic proportions) censuses cover our study period. These were categorised into four categories based on the most recent (based on the census) national average of non-white population defined at cut points of below or equal to (i) half the national average, (ii) the national average, and (iii) two-fold the national average. Both the deprivation and diversity confounders are time varying as there was multiple ONS releases of the statistics used to derive these over the study period.

The LSOA-level IMD scores from the 2007 and 2010 released and ethnic population estimates from the 2001 census are defined by the 2001 census LSOA boundaries. The LSOA-level IMD from 2015 and 2019 and the ethnic population estimates from the 2011 census are defined by the 2011 census LSOA boundaries. Finally, ethnic population estimates from the 2021 census are defined by the 2021 census LSOA boundaries. To ensure comparability between years, we mapped the statistics based on the 2001 and 2021 census LSOA boundaries to the 2011 census LSOA boundaries using the ONSs look ups (ONS, 2021).

When mapping from the 2001 and 2021 LSOA boundaries to the 2011 boundaries there were some LSOAs newly defined in 2011 that were not present in the others, see Table 4 for a list. For these, we imputed the IMD score and ethnic proportions by taking an average of the neighbouring LSOAs. With the full set of IMD scores and ethnic proportions, we derived the deprivation deciles and diversity categories, respectively.

Table 4: Missing Lower Layer Super Output Areas (LSOAs) when mapping from the 2001 and 2021 census defined LSOA boundaries to the 2011 census defined boundaries.

LSOA11CD	LSOA11NM	Missing from
E01032522	Nottingham 028F	2001 Census
E01032540	Hartlepool 003G	2001 Census
E01032608	Leeds 067K	2001 Census
E01033730	Greenwich 035D	2001 Census

Figure 3 presents the deprivation deciles and diversity categories for each of the different ONS releases mapped onto the 2011 defined LSOA boundaries.

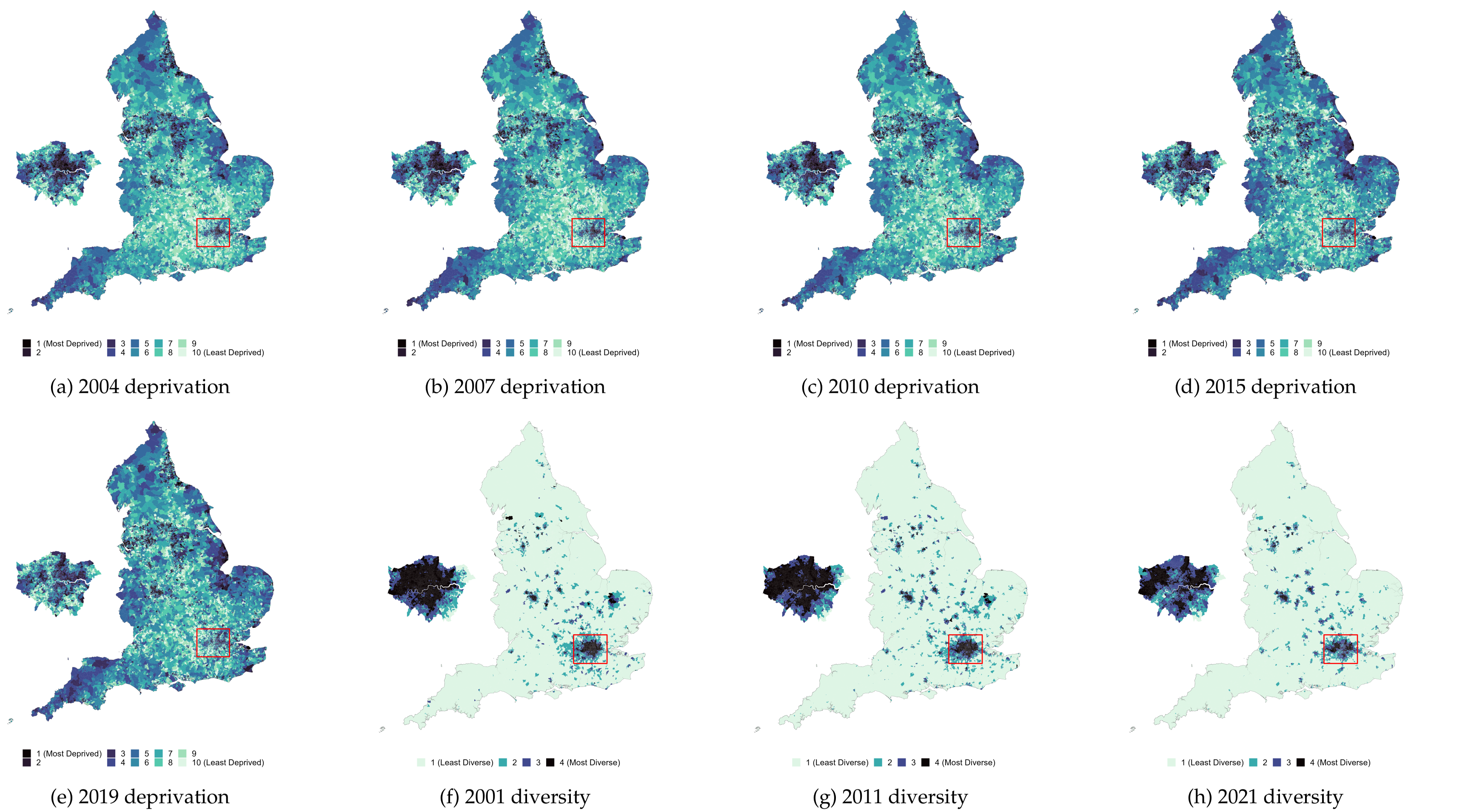

Figure 3: Spatial plots for the deprivation deciles and diversity categories for the different Office for National Statistics releases. The light-to-dark colouring represent changes from the least-to-most deprived and diverse, respectively. For all plots, London is shown separately.

4 Model selection

4.1 Unstructured and structured random effects

In the manuscript, the terms to account for the stratification and clustering of the data structure, $\psi_{p(i)}$ and $\kappa_{k(i)}$, respectively, and the term to account for the individual, ϵ_i, are included in as unstructured random effects which will not change. Therefore, we will not include them in the model selection process. For γ_t and δ_l, the temporal and spatial random effects, respectively, we perform a model selection on these.

When the spatial and temporal random effects are unstructured, they are modelled by zero mean Gaussian distributions with their own specific variances,

$$\gamma_t \sim \mathrm{N}\left(0, \sigma_\gamma^2\right) \quad \text{and} \quad \delta_{s(i)} \sim \mathrm{N}\left(0, \sigma_\delta^2\right).$$

The structure we imposed on the temporal random effect was a random walk of order one (RW1) autoregressive prior. The RW1 prior imposes a local smoothing such that years that are next to one another are more closely correlated than years that are further from one another. For $t = 1, \ldots, T$ yearly time points, the RW1 prior can be written,

$$\begin{aligned} f\left(\gamma_t\right) | \sigma_\gamma^2 &\propto \sigma_\gamma^{-(T-1)} \exp\left(-\frac{\sigma_\gamma^{-2}}{2} \sum_{t=1}^{T-1} \left(\gamma_{t+1} - \gamma_t\right)^2\right) \\ &= \sigma_\gamma^{-(T-1)} \exp\left(-\frac{1}{2} \gamma_t' \boldsymbol{Q}_\gamma \gamma_t\right) \end{aligned}$$

with $\boldsymbol{Q}_\gamma$, the precision matrix which depends on the unknown variance parameter σ_γ^2, has rank $T-1$ and is of the form

$$\boldsymbol{Q} = \sigma_\gamma^{-2} \begin{pmatrix} 1 & -1 & & & & & \\ -1 & 2 & -1 & & & & \\ & -1 & 2 & -1 & & & \\ & & \ddots & \ddots & \ddots & & \\ & & & -1 & 2 & -1 & \\ & & & & -1 & 2 & -1 \\ & & & & & -1 & 1 \end{pmatrix}. \tag{1}$$

The structure we imposed on the spatial random effects accounts for residual confounding at the LSOA level and was specified through the combination of a standard random effect, and an autoregressive structure based on local correlation among neighbouring LSOAs,

$$\delta_{s(i)} = v_{s(i)} + u_{s(i)}$$

where $v_{s(i)} \sim_{\text{iid}} \mathrm{N}(0, 1)$ and $u_{s(i)}$ is modelled with $u_{s(i)} \sim \mathrm{N}\left(0, \sigma_u^2\right)$ for some standard deviation σ_u^2. Modelling residual spatial variation in this manner (modelling a spatial random effect using both an unstructured and structure term) is known as the Besag-York-Mollie (BYM) model (Besag et al., 1991), and is common in many epidemiological settings. Following Riebler et al. (2016), we use the so-called BYM2 parameterization to model both the structured and unstructured spatial effects together,

$$\delta_{s(i)} = \sigma_\delta \left(\sqrt{\phi} u_{s(i)}^* + \sqrt{1-\phi} v_{s(i)}^*\right)$$

where σ_δ^2 controls the marginal variance controlling the weighted sum of $u_{s(i)}^*$ and $v_{s(i)}^*$, the structured and unstructured spatial effects which are standardised to have the total variance sum to one. In addition, $\phi \in [0, 1]$ is the mixing parameter that measures the proportion of the marginal variance explained by the structured spatial effect $u_{s(i)}^*$.

4.2 Hyperpriors

For all the parameters of the random effects, we set the hyper-priors to be penalized complexity (PC) priors (Simpson et al., 2017). PC priors are designed to penalise deviations in the posterior distribution from a simpler, "base"

model. PC priors were developed as a solution to the problem that arises when a prior forces overfitting (when it is impossible to distinguish between a model that is supported by the data or by a poor prior choice). For ζ, a flexibility parameter controlling how much the model results depend on the data and the prior, $\mathcal{Q}(\zeta)$ is an interpretable transformation (i.e., standard deviation, precision) of ζ, U a "sensible" upper bound and p the probability of ζ being in the upper bound; a PC prior is specified

$$\Pr\left(\mathcal{Q}(\zeta) > U\right) = p.$$

All the standard deviations parameters for the stratification, clustering, temporal and spatial random effects have a PC prior where $U = 1$ and $p = 0.01$. Additionally, the spatial effect mixing parameter, ϕ, has a $\Pr(\phi > 1/2) = 2/3$ PC prior. The larger p for the mixing parameter reflects the prior belief for more of the spatial variation to be described by the structured spatial effect.

4.3 Results

In the Manuscript, we included structured temporal and spatial random effects to account for residual confounding in time and space. As part of the analysis, we performed a model selection process (starting from a 'base' model of only the fixed effects) to consider the inclusion of the temporal and spatial random effects as well as all combinations of unstructured and structured temporal and spatial random effects.

The results of the model selection are in Table 5. We evaluated the model selection using the Deviance information criterion (DIC; Spiegelhalter et al., 2002) and the Watanabe-Akaike information criterion (WAIC; Watanabe and Opper, 2010). Both the DIC and the WAIC provides a score based on a combination of model fit and model complexity and are commonly used for comparing model fits from a Bayesian hierarchical framework. In addition, we included an alternative scoring rule, the Interval Score (IS; Gneiting and Raftery, 2007). The DIC and WAIC are likelihood-based scores, and the IS is a distribution based scored that provides a score based on penalising if the fitted values are outside of the uncertainty interval. For all scores, the scale is not important, but comparing the relative change within each score is, and the lower the score the better fitting the model.

For the DIC and WAIC score, the model with an unstructured temporal and structured spatial effect scored the best. For the IS score, the model with the structured temporal and spatial effect scored the best. In general, the base model scored the worst for all scores and including a spatial term only scores better than including a temporal term only in addition to the base model. Include both temporal and spatial terms produce models with better scores compared to those with no random effects or one of the random effects. The relative difference between all scores for the model with an unstructured temporal and structured spatial and the model with a structure temporal and structured spatial is little. We note the structured temporal and unstructured spatial random effects model has better scores in comparison to the model chosen, structured time and space, but the difference is marginal and we trade the slightly better score for the additional insight offered by the spatial mixing parameter, ϕ.

Table 5: Model selection for the inclusion of and type of the temporal and spatial random effect. The lower the WAIC score, the better performing the model. The model selected for the Manuscript is in ***emphasised text***.

Temporal Random Effect	Spatial Random Effect	DIC	WAIC	IS	Time
-	-	1332908.84	1334511.47	42.68	199
Unstructured	-	1332760.02	1334359.44	42.58	236
Structured	-	1332760.78	1334340.41	42.57	242
-	Unstructured	1330179.27	1332990.74	41.49	1270
-	Structured	1332545.45	1334253.56	42.51	10117
Unstructured	Unstructured	1332544.49	1334173.89	42.43	935
Unstructured	Structured	1332439.79	1334137.73	42.38	17738
Structured	Unstructured	1330869.98	1332664.62	40.90	2382
Structured	***Structured***	***1332430.44***	***1334109.88***	***42.43***	***11362***

5 Final model results

The summary statistics for all the selected model parameters (fixed and random effects) are in Table 6.

Table 6: Final model parameters.

	**Parameter (95% Credible Interval)**
**Control**	
Intercept	10.7369 (9.5935, 11.8804)
Time	0.0099 (-0.0142, 0.0339)
Intervention	-0.0227 (-0.287, 0.2417)
$Time^+$	-0.0004 (-0.002, 0.0011)
**Difference**	
Intercept	2.1607 (1.9734, 2.348)
Time	0.0026 (0, 0.0052)
Intervention	0.5413 (0.1214, 0.9612)
$Time^+$	-0.0011 (-0.0019, -0.0003)
**Age: Ref. [16, 25)**	
[25, 35)	0.5751 (0.4864, 0.6639)
[35, 45)	0.8239 (0.7247, 0.9231)
[45, 55)	0.8534 (0.7488, 0.9581)
[55, 65)	0.3467 (0.2317, 0.4618)
**Education: Ref. Below GCSE and other**	
GCSE, A-level or equivalent	-0.1372 (-0.2363, -0.0382)
Degree or higher	-0.2363 (-0.3359, -0.1367)
**Ethnicity: Ref. White**	
Asian	-0.5346 (-0.6653, -0.4038)
Black	-1.2655 (-1.4358, -1.0953)
Mixed	0.0456 (-0.1926, 0.2839)
Other	-0.425 (-0.6238, -0.2262)
**Relationship: Ref. Single**	
Relationship	-0.5899 (-0.6593, -0.5206)
**Sex: Ref. Male**	
Female	1.2826 (1.2059, 1.3594)
**Deprivation: Ref 10 (Least Deprived)**	
1 (Most Deprived)	0.6456 (0.5035, 0.7877)
2	0.574 (0.4391, 0.709)
3	0.405 (0.2728, 0.5372)
4	0.3942 (0.2653, 0.523)
5	0.2671 (0.1393, 0.3948)
6	0.2679 (0.143, 0.3929)
7	0.1971 (0.0723, 0.3219)
8	0.1467 (0.0253, 0.2681)
9	0.0431 (-0.0723, 0.1586)
**Diversity: Ref. 1 (Least Diverse)**	
2	0.1333 (0.0484, 0.2181)
3	0.1707 (0.0707, 0.2708)
4 (Most Diverse)	0.0586 (-0.0571, 0.1742)
**Random Effects:**	
Gaussian precision	0.0608 (0.0604, 0.0611)
Strata precision	54.633 (44.8536, 64.4781)
Cluster precision	44.5959 (38.3227, 53.2525)
Temporal precision	52.8754 (46.0793, 60.6436)
Spatial precision	16.0706 (13.7177, 17.8339)
Spatial mixing	0.0449 (0.0377, 0.0516)
Individual precision	0.0801 (0.0785, 0.0813)

Figure 4 presents the fixed effects with their lower and upper uncertainty intervals.

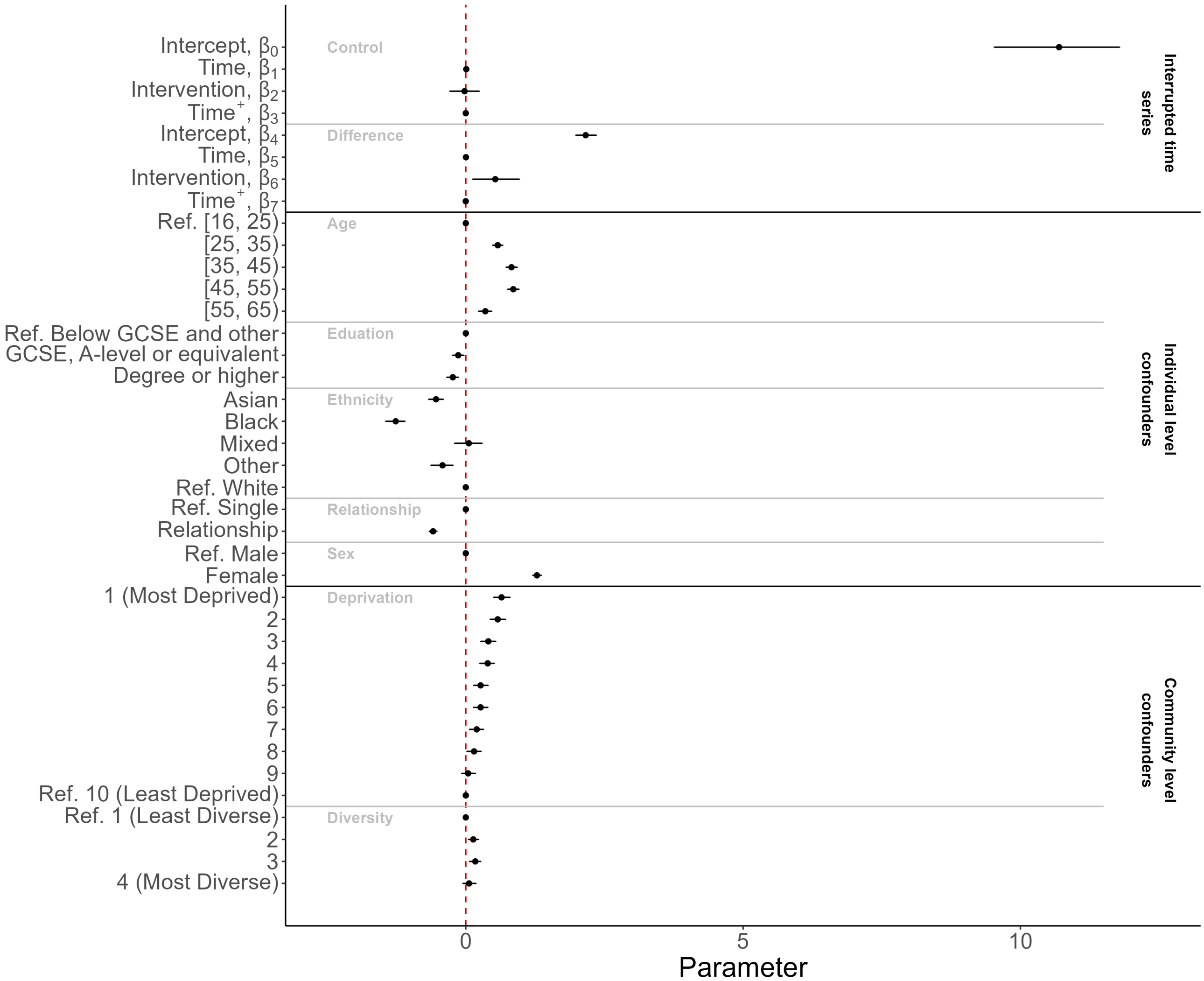

Figure 4: Final model fit fixed parameters.

Figure 5 presents the residual plots of the temporal (left) and spatial (right) random effects. As expected, there is more variation in the earlier and later months in the temporal plot, Figure 5a, as these months are seen the least in the study period.

The estimated value for the mixing parameter, ϕ (the parameter which attributes the amount of residual spatial variation tat is due to an unstructured and structure random effect), is 0.0449 (95% CrI 0.0377 – 0.0516) indicating the majority of the residual spatial variation is unstructured. Figure 5b reflects this with some areas being more similar (mainly within cities with larger populations) but the majority of the areas in England having very little residual spatial variation.

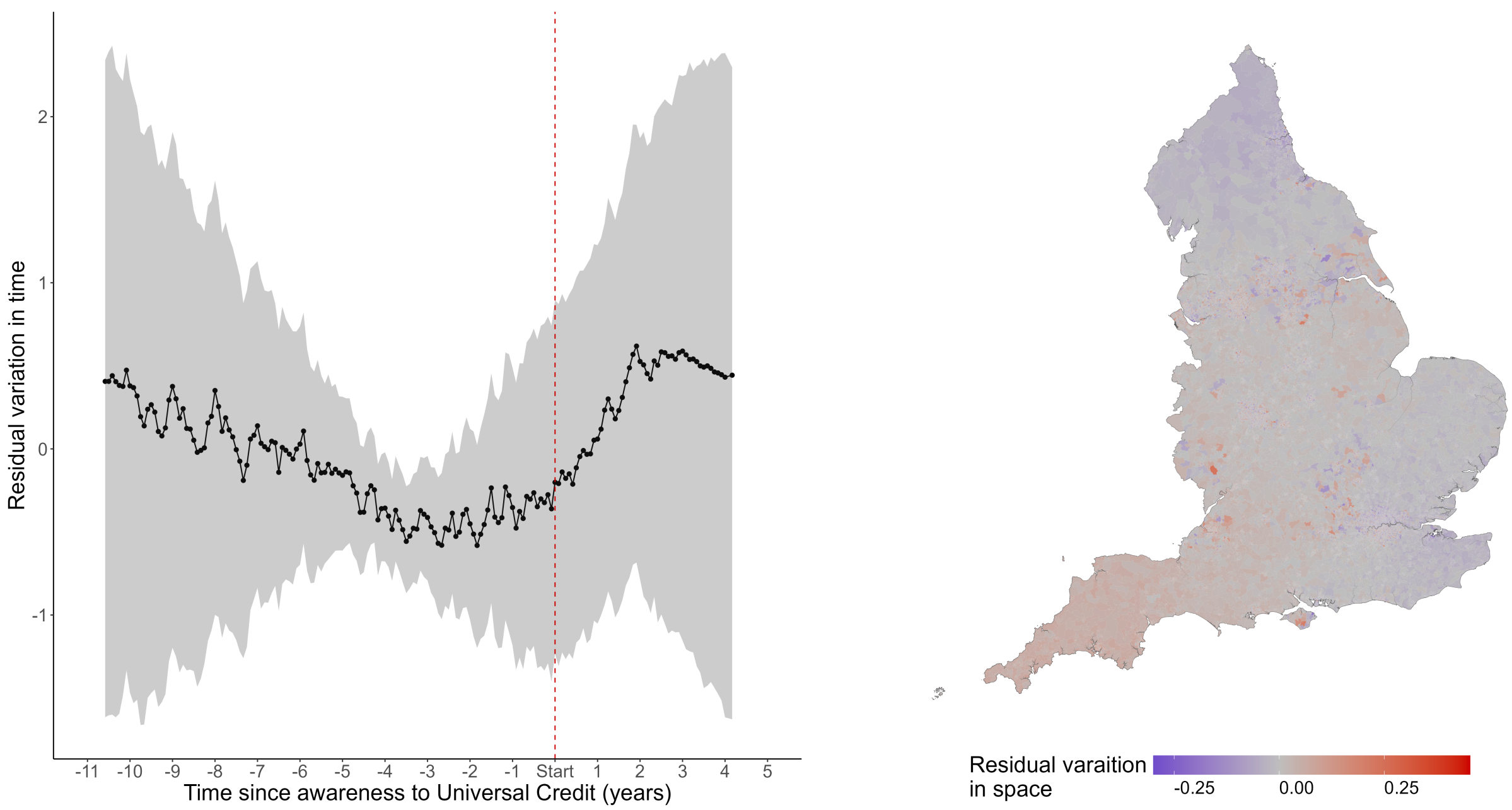

(a) Temporal.

(b) Spatial.

Figure 5: Random effect residual plots.

6 Sensitivity analysis

We defined the start of the intervention as when an LTLA becomes "contextually aware" of UC. A contextual awareness is when enough people within a LTLA are aware of UC that even if an individual is not on UC, their mental well-being could be potentially influenced it. We used a contextual awareness to UC instead of being 'introduced' to UC (Wickham et al., 2020); when at least one person in a LTLA is receiving UC. In the Manuscript, we defined an LTLA to be contextually aware when the number of individuals receiving UC reached 25% of the number of individuals on UC in the study periods last monthly total. From the Department of Work and Pensions (DWP) (DWP, 2022), the month we used was December 2021. We performed a sensitivity analysis to see how robust the results are to difference in changes to the definition of the intervention. We compared the intervention being defined by an Introduction or contextual awareness.

Figure 6 presents the results of the sensitivity analysis for the parameters of the confounders (The parameters for the ITS terms are in the main Manuscript), and Table 7 presents the results for all parameters in the models. The main takeaway is the interpretation for the majority of the parameters is robust to the definition of the intervention.

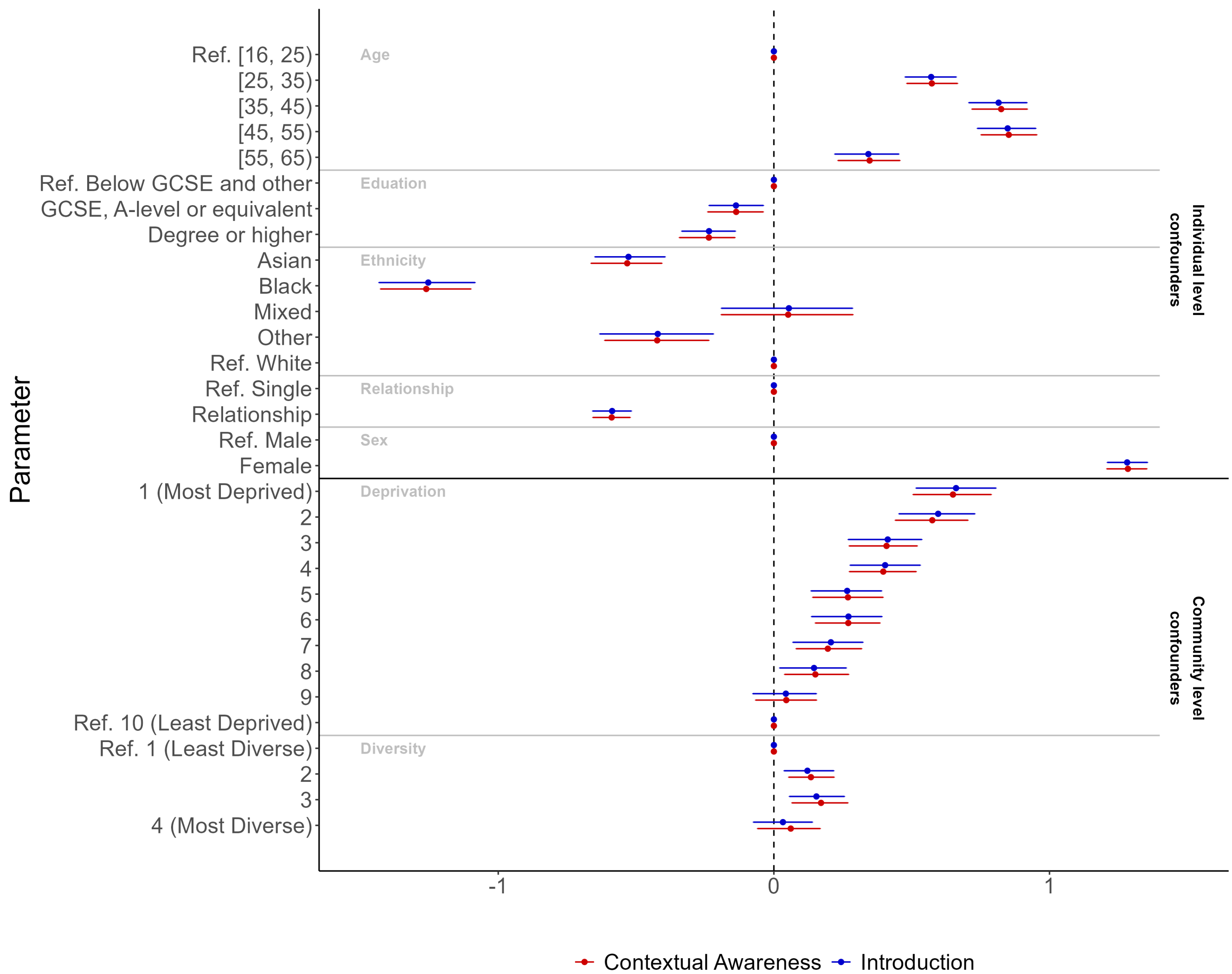

Figure 6: Sensitivity analysis for the definition of when the intervention occurs. The results in red and blue are when the intervention is defined by an introduction or a contextual awareness, respectively. Each facet is one of the confounders with the different levels along the x-axis.

Table 7: Sensitivity analysis for the definition of contextual awareness to Universal Credit. The first value is the point estimate of the median from the posterior distribution and the values in the brackets are the 95% Credible Intervals.

	Introduction	Contextual Awareness
**Control**		
Intercept	10.5202 (9.5729, 11.4674)	10.7369 (9.5935, 11.8804)
Time	0.0209 (-0.0167, 0.0585)	0.0099 (-0.0142, 0.0339)
Intervention	0.0623 (-0.1938, 0.3183)	-0.0227 (-0.287, 0.2417)
$Time^{+}$	-0.0009 (-0.0023, 0.0005)	-0.0004 (-0.002, 0.0011)
**Difference**		
Intercept	1.9488 (1.7171, 2.1805)	2.1607 (1.9734, 2.348)
Time	0.0025 (-0.0057, 0.0107)	0.0026 (0, 0.0052)
Intervention	0.186 (-0.1061, 0.478)	0.5413 (0.1214, 0.9612)
$Time^{+}$	-0.0001 (-0.0004, 0.0002)	-0.0011 (-0.0019, -0.0003)
**Age: Ref. [16, 25)**		
[25, 35)	0.5711 (0.4823, 0.6599)	0.5751 (0.4864, 0.6639)
[35, 45)	0.8175 (0.7182, 0.9168)	0.8239 (0.7247, 0.9231)
[45, 55)	0.848 (0.7433, 0.9527)	0.8534 (0.7488, 0.9581)
[55, 65)	0.3403 (0.2252, 0.4554)	0.3467 (0.2317, 0.4618)
**Education: Ref. Below GCSE and other**		
GCSE, A-level or equivalent	-0.1387 (-0.2378, -0.0396)	-0.1372 (-0.2363, -0.0382)
Degree or higher	-0.2375 (-0.3372, -0.1379)	-0.2363 (-0.3359, -0.1367)
**Ethnicity: Ref. White**		
Asian	-0.5267 (-0.6574, -0.3959)	-0.5346 (-0.6653, -0.4038)
Black	-1.267 (-1.4372, -1.0967)	-1.2655 (-1.4358, -1.0953)
Mixed	0.0507 (-0.1876, 0.2889)	0.0456 (-0.1926, 0.2839)
Other	-0.4275 (-0.6263, -0.2287)	-0.425 (-0.6238, -0.2262)
**Relationship: Ref. Single**		
Relationship	-0.5882 (-0.6576, -0.5188)	-0.5899 (-0.6593, -0.5206)
**Sex: Ref. Male**		
Female	1.2808 (1.204, 1.3576)	1.2826 (1.2059, 1.3594)
**Deprivation: Ref. 10 (Least Deprived)**		
1 (Most Deprived)	0.6673 (0.5252, 0.8094)	0.6456 (0.5035, 0.7877)
2	0.5932 (0.4582, 0.7283)	0.574 (0.4391, 0.709)
3	0.4113 (0.2791, 0.5436)	0.405 (0.2728, 0.5372)
4	0.4058 (0.2769, 0.5348)	0.3942 (0.2653, 0.523)
5	0.2699 (0.142, 0.3977)	0.2671 (0.1393, 0.3948)
6	0.2697 (0.1446, 0.3947)	0.2679 (0.143, 0.3929)
7	0.2063 (0.0814, 0.3312)	0.1971 (0.0723, 0.3219)
8	0.1468 (0.0253, 0.2683)	0.1467 (0.0253, 0.2681)
9	0.0423 (-0.0732, 0.1578)	0.0431 (-0.0723, 0.1586)
**Diversity: Ref. 1 (Least Diverse)**		
2	0.1219 (0.0369, 0.2069)	0.1333 (0.0484, 0.2181)
3	0.1542 (0.0539, 0.2544)	0.1707 (0.0707, 0.2708)
4 (Most Diverse)	0.03 (-0.0857, 0.1458)	0.0586 (-0.0571, 0.1742)
**Random Effects:**		
Gaussian precision	0.0607 (0.0604, 0.0611)	0.0608 (0.0604, 0.0611)
Strata precision	56.0621 (51.1449, 61.1836)	54.633 (44.8536, 64.4781)
Cluster precision	45.0069 (40.9188, 49.7468)	44.5959 (38.3227, 53.2525)
Temporal precision	43.7821 (40.2238, 47.8762)	52.8754 (46.0793, 60.6436)
Spatial precision	16.1903 (14.7015, 17.5099)	16.0706 (13.7177, 17.8339)
Spatial mixing	0.0413 (0.038, 0.0447)	0.0449 (0.0377, 0.0516)
Individual precision	0.0795 (0.0767, 0.0811)	0.0801 (0.0785, 0.0813)

References

J. Besag, J. York, and A. Mollié. Bayesian image restoration, with two applications in spatial statistics. *Annals of the institute of statistical mathematics*, 43:1–20, 1991.

DWP. Stat-Xplore. Technical report, Department for Work and Pensions, 2022. URL https://stat-xplore.dwp.gov.uk. Accessed May 2022.

T. Gneiting and A. E. Raftery. Strictly proper scoring rules, prediction, and estimation. *Journal of the American Statistical Association*, 102(477):359–378, 2007.

ONS. Ethnic group, England and Wales: Census 2001. Technical report, Office for National Statistics, 2001a. URL https://www.nomisweb.co.uk/datasets/ks006. Accessed January 2024.

ONS. Ethnic group, England and Wales: Census 2021. Technical report, Office for National Statistics, 2001b. URL https://www.ons.gov.uk/peoplepopulationandcommunity/culturalidentity/ethnicity/bulletins/ethnicgroupenglandandwales/census2021. Accessed January 2024.

ONS. English indices of deprivation 2007. Technical report, Office for National Statistics, 2007. URL https://webarchive.nationalarchives.gov.uk/ukgwa/20100411141238/http://www.communities.gov.uk/communities/neighbourhoodrenewal/deprivation/deprivation07/. Accessed January 2024.

ONS. English indices of deprivation 2010. Technical report, Office for National Statistics, 2011. URL https://www.gov.uk/government/statistics/english-indices-of-deprivation-2010. Accessed January 2024.

ONS. English indices of deprivation 2015. Technical report, Office for National Statistics, 2015. URL https://www.gov.uk/government/statistics/english-indices-of-deprivation-2015. Accessed January 2024.

ONS. English indices of deprivation 2019. Technical report, Office for National Statistics, 2019. URL https://www.gov.uk/government/statistics/english-indices-of-deprivation-2019. Accessed January 2024.

ONS. Census 2021 geographies. Technical report, Office for National Statistics, 2021. URL https://www.ons.gov.uk/methodology/geography/ukgeographies/censusgeographies/census2021geographies. Accessed January 2024.

ONS. Ethnic group, England and Wales: Census 2011. Technical report, Office for National Statistics, 2022a. URL https://www.nomisweb.co.uk/census/2011/qs201ew. Accessed January 2024.

ONS. Ethnic group, England and Wales: Census 2021. Technical report, Office for National Statistics, 2022b. URL https://www.ons.gov.uk/datasets/TS021/editions/2021/versions/1. Accessed January 2024.

A. Riebler, S. H. Sørbye, D. Simpson, and H. Rue. An intuitive bayesian spatial model for disease mapping that accounts for scaling. *Statistical methods in medical research*, 25(4):1145–1165, 2016.

D. Simpson, H. Rue, A. Riebler, T. G. Martins, and S. H. Sørbye. Penalising model component complexity: A principled, practical approach to constructing priors. *Statistical Science*, 32:1–28, 2017.

D. J. Spiegelhalter, N. G. Best, B. P. Carlin, and A. Van Der Linde. Bayesian measures of model complexity and fit. *Journal of the Royal Statistical Society Series B: Statistical Methodology*, 64(4):583–639, 2002.

S. Watanabe and M. Opper. Asymptotic equivalence of Bayes cross validation and widely applicable information criterion in singular learning theory. *Journal of Machine Learning Research*, 11(12):3571–3594, 2010.

S. Wickham, L. Bentley, T. Rose, M. Whitehead, D. Taylor-Robinson, and B. Barr. Effects on mental health of a UK welfare reform, Universal Credit: a longitudinal controlled study. *The Lancet Public Health*, 5(3):e157–e164, 2020.
